# Nitrate removal study of synthesized nano γ-alumina and magnetite-alumina nanocomposite adsorbents prepared by various methods and precursors

**DOI:** 10.1038/s41598-024-58459-z

**Published:** 2024-04-01

**Authors:** Maasoumeh Khatamian, Saeedeh Khadivi Derakhshan, Shamin Hosseini Nami, Sara Fazli-Shokouhi

**Affiliations:** 1 Department of Inorganic Chemistry, Faculty of Chemistry, University of Tabriz, Tabriz, 5166616471 Iran; 2 School of Chemical, Biological, and Materials Engineering, The University of Oklahoma, Norman, OK 73019 USA; 3 Faculty of Materials Engineering, Sahand University of Technology, Tabriz, 513351996 Iran

**Keywords:** γ-Al_2_O_3_, Fe_3_O_4_, Nanoparticle, NO^3−^, Water treatment, Environmental chemistry, Inorganic chemistry, Materials chemistry, Chemical synthesis

## Abstract

The challenges in water treatment include the need for efficient removal of pollutants like nitrate, which poses significant environmental and health risks. Alumina's significance lies in its proven effectiveness as an adsorbent for nitrate removal due to its high surface area and affinity for nitrate ions. This study delves into the synthesis of differen nano-sized γ-alumina (γA1-5) employing diverse precursors and methods, including nepheline syenite, lime, aluminum hydroxide, precipitation, and hydrothermal processes at varying reaction times. Simultaneously, magnetite (Fe_3_O_4_) nanoparticles and magnetite/γ-alumina nanocomposites (F_n_/γA5) were synthesized using the co-precipitation method with varying weight ratios (n). Our primary objective was to optimize γ-alumina synthesis by comparing multiple methods, shedding light on the influence of different precursors and sources. Hence, a comprehensive adsorption study was conducted to assess the materials’ efficacy in nitrate removal. This study fills gaps in the literature, providing a novel perspective through the simultaneous assessment of magnetite/alumina nanocomposites and pure alumina performance. Structural and morphological properties were studied employing XRD, FT-IR, FESEM, EDX, XRD, and VSM techniques. The conducted experiments for γA5, F_5_/γA5, and F_10_/γA5 nanocomposites showcased the optimum pH of 5 and contact time of 45 min for all samples. The influence of nitrate’s initial concentration on the removal percentage was investigated with initial concentrations of 10 ppm, 50 ppm, and 100 ppm. γA5, F_5_/γA5 and F_10_/γA5 nanocomposites had 17.3%, 55%, and 70% at 10 ppm, 18%, 55.16%, and 74% at 50 ppm, and 8.6%, 53.1%, and 63%, respectively. The results highlighted that F_10_/γA5 can be used as a remarkable adsorbent for wastewater treatment purposes.

## Introduction

The escalating prevalence of nitrate in aqueous environments has engendered heightened concerns regarding both human and animal well-being, alongside ecological perturbations. The inherent characteristics of nitrate, typified by its aqueous solubility and minimal propensity for immediate soil binding, render it predisposed to facile runoff and migration within water systems. Nitrate has thus emerged as a significant contaminant in groundwater and industrial effluents^1^, particularly within the milieu of developing nations. Consequently, the imperative to expeditiously investigate methodologies for the removal of these ions from water effluents has become a preeminent priority, in order to safeguard the reservoirs of the future^2–5^. For this purpose, multiple methods have been applied and investigated such as reverse osmosis^6^, ion exchange^7^, and chromatography^8^. The above-mentioned methods have extortionate costs and complexity; thus, other alternatives such as adsorption^9^, chemical methods^10^, biological methods^11^, and nanotechnology^12^ have been heavily investigated^13–15^.

Among the previously delineated methodologies, adsorption has commanded considerable attention due to its cost-effectiveness, relatively unembellished design, and operational simplicity^16–18^. Conventionally, a spectrum of adsorbents has been discerningly chosen for the explicit purpose of nitrate removal^19–21^. Furthermore, nano-adsorbents have been the subject of meticulous scrutiny, gaining ascendancy owing to their propitious attributes, notably including an expansive surface area, inherent self-assembly prowess, and markedly heightened reactivity ^22–24^. While nanomaterials are commercially procurable^25,26^, their synthesis affords a finer degree of tailoring for requisite special characterizations^27^. Various methodologies, such as mechanical milling, sol–gel processes, hydrothermal modalities, chemical reduction, ultrasound, precipitation, and microwave-assisted preparation, have been invoked in the synthesis of metallic nanoparticles^28–33^. The precipitation method for the synthesis of nanomaterials imparts substantial influence over both particle size and morphology, offering the potential for attaining materials of high purity characterized by remarkable monodispersity. This approach is expeditious and straightforward, minimizing the propensity for aggregation. Alternatively, the hydrothermal route affords precise control over particle size, morphology, and distribution. Employing this methodology enables the production of finely crystallized and homogeneous powders, along with highly crystalline nanocrystals. However, it is imperative to note that experimental parameters such as temperature, precursor type, and reaction time wield considerable influence over the aforementioned material characteristics^30,34–36^.

Among adosbents, metal oxides are favorable regarding the elimination of pollutants due to their cost-effectiveness, thermal resistance, and remarkable mechanical properties^37–41^. Aluminum oxide (Al_2_O_3_) or alumina is one of the important oxides which can be in many crystalline structures such as α-Al_2_O_3_, γ-Al_2_O_3_, etc. Alumina has become the focus of studies for water treatment due to its non-toxicity, chemical stability, large inner surface, and porosity ^42–44^. Hafshejani et al. ^45^ investigated and optimized fluoride removal from water using Al_2_O_3_ nanoparticles. They emphasized the importance of the precursor, solvent, and method of the preparation of the metal nanoparticles and their adsorption capacity. Prabhakar et al.^46^ carried out experiments related to the arsenite removal from groundwater by alumina nanoparticles. They proved that low-cost and easily synthesized nanoparticles can be regenerated effortlessly in order to acquire an economic operation and advantage. The adsorption performance of alumina nanoparticles was investigated by Bhatnagar et al. ^2^; moreover, the conducted study showed that these nanoparticles can be an efficient adsorbent for nitrate removal from water. Chitan et al.^47^ carried out a study that concluded that synthesized nano alumina from nepheline syenite could have higher adsorption capacity for dyes compared to industrial or Merck alumina. Another metal oxide (iron oxide) can result in a compatible, non-toxic and eco-friendly, recyclable, and efficient adsorbent with fast reactivity^48^. Mukhopadhyay et al.^49^ investigated the adsorption capacity of Fe-exchanged materials and iron oxide nanoparticles for nitrate. The acquired data indicated that nitrate removal using both materials was highly pH-dependent. Wiriyathamcharoen and their team^50^ conducted experiments to acquire the efficiency of Fe nanoparticle-based adsorbent. According to the findings of the study, loading of these nanoparticles resulted in materials that had a remarkably high capacity for both phosphate and nitrate; which can be used, recycled, and re-used in industrial and agricultural wastewater treatment.

Considering the comprehensice literature review, previously conducted studies have not compared different methods of alumina synthesis with different precursors and sources to find the most efficient one. Moreover, no studies have been conducted a simultaneous study on the performance of magnetite/alumina nanocomposite with different ratios and pure alumina. Furthermore, most of the studies have focused either on characterization or adsorption. Thus, in the present study magnetite and γ-alumina nanoparticles were successfully synthesized using nepheline syenite, nepheline syenite, lime, and aluminum hydroxide using co-precipitation and hydrothermal methods at different reaction times. All of the synthesized materials were further scrutinized using FESEM, EDX, XRD, VSM, and FT-IR techniques. A series of experiments were conducted using the best nano alumina nanoparticles and their related nanocomposites with magnetite for nitrate removal from water. The removal performance was investigated by altering the various affecting parameters such as pH (3–9), adsorbent dosage (0.05–0.15 g), time (0–60 min), and initial concentration of nitrate (10–100 ppm). Further, the obtained result was fit into appropriate kinetic models.

## Materials and methods

The detailed procedures and methods are elucidated in the Supplementary information (SI) file.

### Preparation of γ-alumina

For preparation of the first γ-alumina (γA1), nepheline syenite ore was utilized as a precursor, with HCl serving as the solvent, boehmite, and NaOH as precipitating agents for impurities in the ore, and ethanol for washing. Initially, nepheline syenite ore was washed with distilled water. Following the filtration and drying process, the obtained sediment was transferred to an electric furnace and heated at 800 °C for 12 h to loosen the bonds of silicon and aluminum. A mixture of nepheline syenite ore and HCl (6 M) in a weight ratio of 1 to 12.5 (1 g to 57.14 ml) was stirred under reflux conditions for 8 h at 70 °C. After filtration, the pH of the resulting solution was adjusted to 9 using NaOH solution (5 M) to precipitate impurities such as silica, iron, sodium, potassium, etc., in the nepheline syenite ore. The orange precipitate was separated by centrifugation, and the pH of the solution was adjusted to 4 using HCl solution. Finally, a gel-like solution was obtained. The obtained gel underwent ultrasonic waves for 15 min and was then left at room temperature for 72 h to complete the aging process and precipitate the boehmite gel. The precipitate was separated by centrifugation and washed with ethanol several times. After drying at ambient temperature, the desired sediment was calcinated at 750 °C for 3 h.

With the aim of synthesizing the second γ-alumina (γA2), nepheline syenite ore and lime were employed as precursors, NaOH acted as the solvent, HNO_3_ functioned as the precipitating agent, and distilled water was utilized for washing. Initially, nepheline syenite ore and lime were meticulously mixed in a 1:2 weight ratio (200 g: 400 g), ensuring a CaO to SiO_2_ ratio approximating 2. To achieve homogeneity, distilled water (30 wt.%) was incorporated into the mixture, which underwent stirring for 4 h, followed by drying in an electric furnace at 1300 °C for 4 h. The acquired powder from the mixture was introduced into a NaOH solution with a ratio of 1:2.5 and subjected to stirring under reflux conditions at 70 °C for 24 h. After the sediment separation through centrifugation, 0.2 g of quicklime was added to 100 ml of the solution and thermally treated in an oven for a specific duration. Following the introduction of HNO_3_ and pH adjustment to 10, the solution underwent a hydrothermal process at 180 °C for 48 h. Subsequent to the reaction, the autoclave was gradually cooled to room temperature. The resulting white sediment, obtained through centrifugation, underwent several washes with distilled water. Lastly, it was dried at 100 °C for 24 h before undergoing calcination at 750 °C for 3 h.

For obtaining γ-alumina from aluminum hydroxide without hydrothermal synthesis (γA3), aluminum hydroxide (Al (OH)_3_) served as the primary precursor, while polyethylene glycol functioned as a spacer. The employed solvent was double-distilled water, and ammonia (NH_3_) acted as the precipitating agent. To initiate the process, 2 g of aluminum hydroxide and 1 g of polyethylene glycol were dissolved in 50 ml of double-distilled water. The pH of the resultant solution was precisely adjusted to 10 by the gradual addition of NH_3_ solution (2 M). Subsequently, the meticulously prepared solution underwent stirring at room temperature for a duration of 5 h. Following the separation and thorough washing of the sediment, the next step involved calcination at 750 °C for a period of 3 h.

For the synthesis of γ-alumina using hydrothermal synthesis (γA4), the identical procedures outlined in γA3 synthesis were meticulously followed to obtain the solution. Upon completion of the stirring process, the solution was transferred to the autoclave and maintained at a temperature of 180 °C for a duration of 48 h. Following the conclusion of the reaction and subsequent cooling of the autoclave to room temperature, the resulting material was separated, subjected to thorough washing, and ultimately calcinated at 750 °C for 3 h.

For preparing γ-alumina with hydrothermal synthesis and extended time (γA5), the identical solution was prepared following the procedures outlined in γA3 synthesis, undergoing stirring at 70 °C for a duration of 7 days. Subsequent to the targeted time period, the resulting sediment was subjected to drying at 100 °C for 24 h, followed by calcination at 900 °C for 3 h.

### Preparation of Fe_3_O_4_ nanoparticles

In this study, Fe_3_O_4_ nanoparticles, prepared through co-precipitation, were synthesized using divalent iron (FeCl_2_·4H_2_O) and trivalent iron (FeCl_3_·6H_2_O) as precursors. The solvent was double-distilled water, and nitrogen gas was utilized for deoxygenation, with NH_3_ serving as the precipitating agent. To initiate the process, 3.3 g of FeCl_2_·4H_2_O and 6 g of FeCl_3_·6H_2_O were introduced into a 500 ml two-hole flask. Subsequently, 30 ml of double-distilled water was added under a nitrogen gas atmosphere. The solution was then heated to 40 °C for 15 min, and the pH was adjusted to 11 using deoxygenated NH_3_ solution.The reaction solution was subjected to reflux conditions under a nitrogen gas atmosphere at 100 °C for 6 h. This led to the formation of black iron oxide nanoparticles with magnetic properties, as represented by Eq. (1). To facilitate nanoparticle washing, the beaker containing the solution was placed on a magnet, and double-distilled water was added and subsequently removed using a syringe. This washing process was iterated several times for thorough purification.

1 $${\text{2 FeCl}}_{{3}} \cdot {\text{6H}}_{{2}} {\text{O }} + {\text{ FeCl}}_{{2}} \cdot {\text{4H}}_{{2}} {\text{O }} + {\text{ 8 NH}}_{{3}} + {\text{ 4 H}}_{{2}} {\text{O}} \to {\text{Fe}}_{{3}} {\text{O}}_{{4}} + {\text{ 8 NH}}_{{4}} {\text{Cl}}$$

### Preparation of magnetite/ γ-alumina nanocomposites (F/ γA)

The nanocomposites were prepared using the solid state diffusion method. For this purpose, 1 g of nano γ-alumina was dispersed in 10 ml of ethanol with the help of ultrasonic waves for 3 h. Subsequently, iron oxide nanoparticles with concentrations 5 wt.%, 10 wt.%, and 30 wt.% were introduced into the suspension, followed by stirring at room temperature for 24 h. 5 wt.%, %10 wt.%, and 30 wt.% coressponded to 1 g of nano γ-alumina in 0.053, 0.11, and 0.43 g of magnetite (Fe_3_O_4_), respectively. The samples were denoted as F_n_/γA, where ‘n’ represents the weight percentage of magnetite nanoparticles in the nanocomposites. The resulting nanocomposites are visually depicted in Supplementary Fig. S.1.

### Nitrate removal and effect of various parameters

In order to prepare a nitrate mother solution with a concentration of 1000 ppm, 0.815 g of oven-dried potassium nitrate was dissolved in 500 ml of distilled water. Solutions with lower concentrations were prepared by increasing the volume of the mother solution. In accordance with established protocols, a solution conforming to standardized procedures was meticulously prepared. Subsequently, the absorbance spectra of the samples were systematically recorded across varying wavelengths (λ) spanning from 190 to 500 nm. The distinctive peak for nitrate absorption manifested at λ_max_ = 210–220 nm, enabling the construction of a calibration curve based on the recorded values (see Supplementary Fig. S.2). Ultimately, the determination of the nitrate solution’s final concentration was executed employing the molar absorption coefficient and Beer–Lambert’s law. This rigorous analytical approach ensures precision and reliability in quantifying nitrate levels within the experimental samples. The molar absorption coefficient of nitrate was calculated using the standard curve, and values were converted to molarity. Considering that the path length is equal to one centimeter, the slope of the standard curve represents the molar absorption coefficient of nitrate, as indicated by Eq. 2, representing the Beer–Lambert Law. Additionally, Eq. 3 was employed for the conversion from ppm to molarity.

2 $${\text{A }} = \, \varepsilon {\text{bc}}$$

3 $${\text{Molarity }} = {\text{ ppm }} \times \, 0.00{1 }/{\text{ molecular weight}}$$

To assess the impact of the initial pH of a nitrate solution on removal efficiency, a solution containing 10 ppm of nitrate, with a volume of 100 ml, was meticulously prepared. Subsequently, 0.1 g of both prepared alumina and nanocomposites were introduced, and the solution’s pH was adjusted to 5 using hydrochloric acid. The temporal influence on the process was explored by employing a 100 ml solution with a 10 ppm nitrate concentration at a pH of 5, with 0.1 g of either alumina or nanocomposites. Sampling was conducted at 15 min intervals to elucidate the effect of contact time.Additionally, the adsorbent dosage was systematically investigated by introducing 0.05, 0.1, and 0.15 g of each adsorbent, with measurements taken at 15-min intervals. The examination of the initial concentration of the nitrate solution on removal efficiency involved the preparation of distinct nitrate solutions with initial concentrations of 10, 50, and 100 ppm. The pH of each solution was adjusted to 5, followed by the introduction of 0.1 g of adsorbent per 100 ml at room temperature. Post-reaction, with sampling intervals of 15 min, residual nitrate levels were measured, enabling the calculation of removal percentages. This systematic approach offers comprehensive insights into the nuanced interplay of pH, contact time, adsorbent dosage, and initial nitrate concentration in the removal process.

### Ethical approval

This research did not cause any harm to human subjects or animals.

## Results and discussion

### Characterization of the prepared nano materiales

The examination of the adsorbents involved a thorough analysis utilizing X-ray diffraction (XRD), referencing ASTM cards for detailed interpretation. Supplementary Fig. S.3a–e presents the XRD profiles of the synthesized alumina nanoparticles. The corresponding crystal d values, chemical formula, and crystal system were determined by cross-referencing with ASTM cards, providing a comprehensive characterization of the crystalline structure of the synthesized alumina nanoparticles in this study.The characteristic peaks of alumina at 2θ = 19°, 31°, 37°, 39°, 46°, 61°, and 67° denoted the (440), (333), (400), (222), (311), (220), and (111) crystal planes of alumina due to JCPDS card NO. 10-042^51^. The XRD pattern of prepared nano γ-alumina from nepheline syenite ore (γA1) is shown in Supplementary Fig. S.3a, which did not match well with the characteristics of the standard sample of γ-alumina (Supplementary Table S.1). The identification of additional peaks at 2θ values of 7.31°, 28.61°, 28.40°, and 58.13° served as conclusive evidence of impurities within the synthesized material. Specifically, the peak at 2θ = 28.61° was attributed to impurities stemming from nepheline syenite ore, while the remaining peaks were indicative of salt impurities formed during the pH adjustment process through the addition of sodium hydroxide and hydrochloric acid. Notably, the alumina index peaks were discerned at 2θ = 31.75°, 45.51°, and 66.28°, with the latter peak exhibiting a comparatively lower intensity. The characteristic of peaks including the angle of diffraction (θ), the full width at half maximum (FWHM), and the intensity of peak (I) are provided in Supplementary Table S.2.The approximate crystal size of γA1was calculated according to Debye–Scherrer’s equation (D = kλ/β cos(θ))^52–55^. In this equation D and K stand for the particle size and the constant equal to 0.94, respectively. The wavelength of the X-ray and full width at half maximum of the diffraction peak are λ and *β*, sequentially. The average crystallite size for γ-alumina (γA1) was calculated as 44.06 nm. Furthermore, the analysis of the XRD pattern indicated that the structure of the prepared γ-alumina was cubic in nature. The XRD pattern related to nano γA2 (Supplementary Fig. S.3b and Supplementary Table S.2) demonstrated a good alignment with characteristics of the standard γ-alumina sample (Supplementary Table S.1). The distinctive diffraction peaks associated with γA2 were observed at 2θ angles of 32.62°, 39.41°, 46.24°, and 66.91°, with specific details provided in Supplementary Table S.2. The average crystallite size for γA2 was determined to be 10.95 nm through the application of Debye–Scherrer’s equation. Additionally, the nanostructure of γ-alumina denoted as γA2 exhibited a cubic configuration. Comparing the XRD pattern of γA3 (Supplementary Fig. S.3c and Supplementary Table S.2) to standard samples (Supplementary Table S.1) it was deduced that the synthesized sample did not match due to the low intensity of peak at 2θ = 67.55°. The frequent peaks within the 2θ = 31°–46° range indicated that the crystallization process for the formation of γ-alumina was not completed; hence, further, in this work, the reaction was carried out using a hydrothermal process. The structure of γA3 was cubic and the average crystallite size was 7.27 nm. The X-ray diffraction pattern of the γA4, provided in Supplementary Fig. S.3d and Supplementary Table S.2, did not match well with the specifications of the standard γ-alumina sample. This can be justified by the low intensity of peaks at 2θ = 22.94°, 42.41°, 45.81°, and 67.30°, which indicated the incomplete crystallization process. Thus, the initial temperature, reaction time, and calcination temperature was increased further in this work. The average crystallite size of cubic γA4 was found to be 11.69 nm using. The obtained XRD results for γA5 (Supplementary Fig. S.3e and Supplementary Table S.2) proved that the synthesized nanoparticle matched well considering the standard γ-alumina sample. The characteristic peaks of alumina were detected at 2θ = 32.12°, 39.60°, 45.63°, and 67.19° and the average crystallite size of these cubic nanoparticles was calculated as 16.51 nm. The obtained XRD spectra and results for γA3, γA4, and γA5 indicated that increasing the initial temperature and reaction resulted in nano γ-alumina with desired purity and crystallization.

Due to the obtained spectra for γA1 and its impurities, the rest of the samples were further scrutinized using the FT-IR technique. Supplementary Fig. S.4a–d demonstrates provided FT-IR spectra for γA2, γA3, γA4, and γA5. Supplementary Fig. S.4a illustrates the FT-IR spectrum corresponding to the nano γ-alumina derived from nepheline syenite ore and lime, denoted as γA2. The discernible peaks within the range of 422.95–1028.71 cm^−1^ were attributed to the stretching and bending vibrations of aluminum and oxygen bonds. Additionally, bending vibrations related to the O–H bond in aluminum hydroxide, along with signals indicative of alumina formation, were evident at 1420.75–1520.08 cm^−1^. Bending and stretching vibrations of O–H bonds were further identified at 1642.40–1740.94 cm^−1^ and 3440.93–3847.47 cm^−1^, respectively^56^. For nano γ-alumina without undergoing a hydrothermal process, designated as γA3 and depicted in Supplementary Fig. S.4b, the stretching and bending bonds of Al–O bonds were observable within the range of 611.49–669.18 cm^−1^^57^. The observed peak at 1461.76 cm^−1^ was assigned to the bending vibration of the O–H bond in alumina^57^. The relatively low intensity of the peak associated with alumina formation suggested insufficient time for achieving preferable crystallinity. Additionally, the bending and stretching vibrations of the hydroxyl bond in water were discernible at 1646.01 cm^−1^ and 3449.67 cm^−1^, respectively^58^. These findings provided valuable insights into the molecular characteristics and structural aspects, indicating the temporal considerations for optimal crystalline development. The synthesized nano γ-alumina from aluminum hydroxide using the hydrothermal method (γA4) was scrutinized by FT-IR (Supplementary Fig. S.4c). The vibration of stretching and bending Al–O bond and bending O–H bond of alumina formation appeared at 611.20 cm^−1^ and 1461.54 cm^−1^, sequentially. As it was depicted, the time and crystallinity were not enough due to the low intensity. Assigned peaks to bending and stretching bonds of water molecules were at 1646.49 cm^−1^ and 3452.49 cm^−1^, respectively. Supplementary Fig. S.4d showcases the FT-IR spectrum of γA5. The vibrational analysis revealed stretching and bending bonds of Al–O within the 580.34–832.06 cm^−1^ range. Noteworthy peaks in the 1418.45–1520.04 cm^−1^ range were associated with the bending vibration of the O–H bond during alumina formation^56,57^. The heightened intensity of these peaks affirmed the adequacy of the reaction duration, indicating the successful synthesis of a crystalline structure. Additionally, bending vibrations of O–H bonds in water molecules were identified in the range of 1647.75–1740.97 cm^−1^, with stretching bonds observed within the 3449.62–3742.32 cm^−1^ range. Vibration bonds attributed to ethylene glycol were evident in the 2800–3000 cm^−1^ range across all examined samples, with a notable decrease in intensity observed for γA5^58,59^. This observation suggested that an increase in the initial temperature, reaction time, and calcination temperature led to the completion of the reaction, resulting in the complete combustion of ethylene glycol during elevated calcination temperatures. Furthermore, samples prepared over different durations, including 1 h, 48 h, and 5 days, underwent FT-IR analysis. However, the presented sample exhibited the most favorable results, underscoring the optimal conditions for achieving the desired structural characteristics.

**Figure 1:**
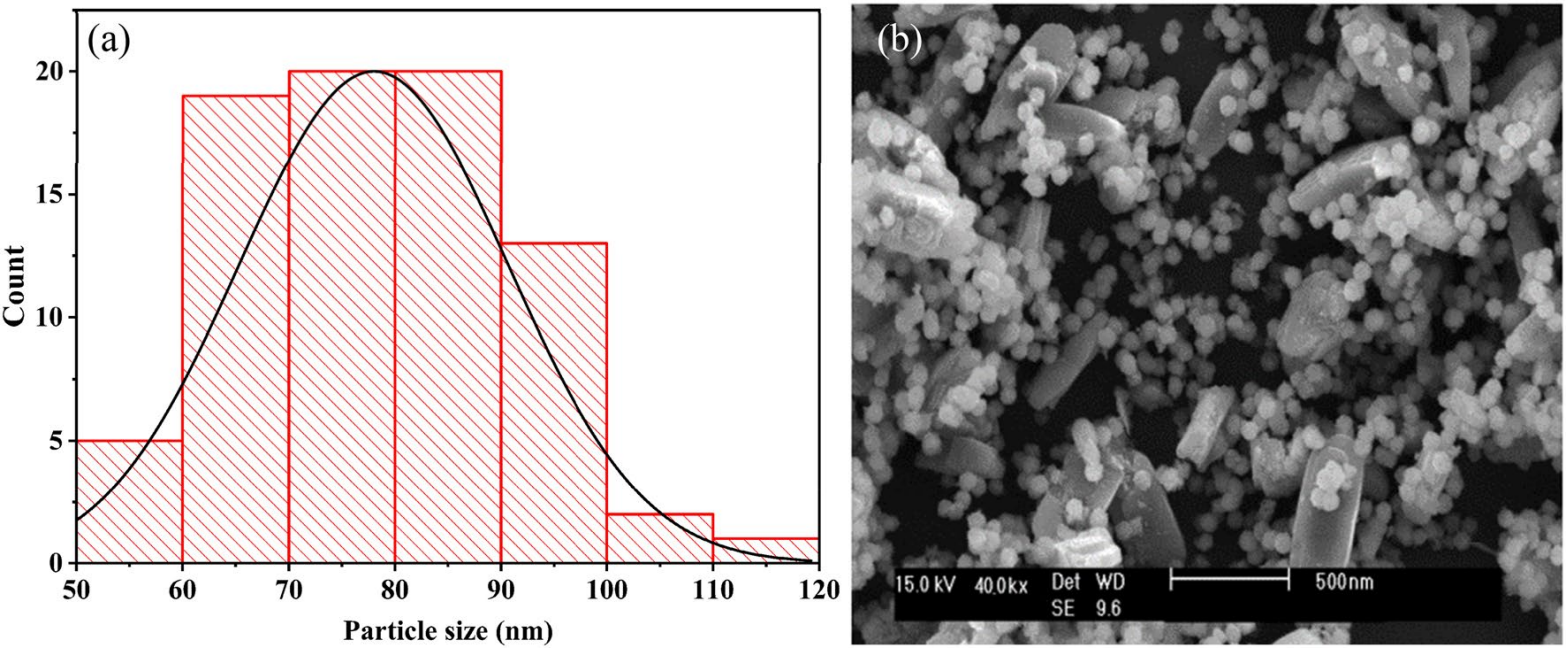
(**a**) Histogram graph, and (**b**) SEM image of γA5.

The conducted characterizations showed that nano γ-alumina from aluminum hydroxide with extended time was better than the other samples; hence, γA5 was further investigated using SEM to scrutinize the morphology of the surface and the size of the alumina particles (using histogram graph)^60,61^. As can be seen in Fig. 1a, the average size of alumina particles was 75.49 nm, with the smallest and largest particles being 52.533, and 113.039 nm, respectively. The crystalline size obtained from XRD was 16.51 nm. According to the Fig. 1b, the prepared alumina had two types of morphology and the increased temperature caused the accumulation of particles and the formation of larger particles. The specific surface area for alumina obtained from nepheline syenite ore and lime was equal to 64.98 m^2^/g.

### Characterization of nanocomposites

F_n_/γA5 nanocomposites were further scrutinized using XRD and the obtained patterns are provided in Supplementary Fig.S.5a–c. The characteristic peaks of alumina X-ray diffraction patterns of F_5_/γA5 (Supplementary Fig.S.5c), and F_10_/γA5 (Supplementary Fig.S.5b) nanocomposites, indicated the stability of the crystalline structure of alumina for these materials. The intensity of the peak at 2θ = 67.20° was higher for F_5_/γA5 compared to F_10_/γA5, due to the reduced iron content. It was also noteworthy that no shift was detected regarding the alumina characteristic peak of the γA5 sample. Although the intensity of alumina-related peaks decreased by increasing the iron content of the nanocomposite, iron oxide’s characteristic peaks for all nanocomposites were all detected at 2θ = 35.64°, 35.57°, and 35.59° for F_30_/γA5, F_10_/γA5, and F_5_/γA5, respectively. Also, these peaks indicated the high crystallinity of the synthesized iron oxide, which increased with the addition of iron oxide amount in the magnetite-alumina composites. The information about the d values and XRD characteristics of Fn/γA5 nanocomposites are given in Supplementary Table S.3. According to the obtained values, the displacements in the d values of nanocomposites indicated the presence of iron oxide nanoparticles on the alumina surface. The broad diffraction pattern in the samples was probably due to the nano size of the prepared nanocomposite. The approximate crystallite size of nanocomposites was calculated using the Debye–Scherer equation^62^.

**Figure 2:**
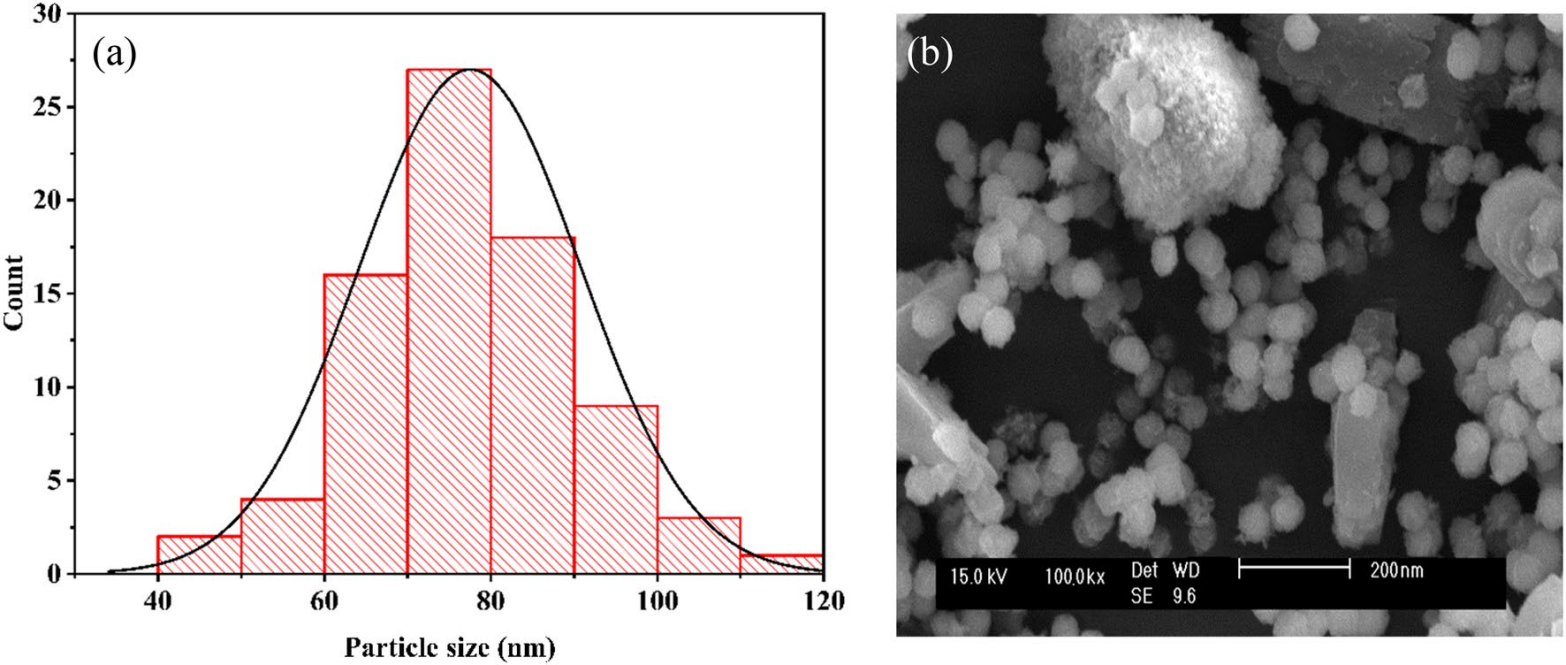
(**a**) Histogram graph, and (**b**) SEM image of F_10_/γA5.

A Fig. 2b shows the SEM image of the F_10_/γA5 nanocomposite which showed the distribution and dispersion of iron oxide nanoparticles on the nano γ-alumina surface. The obtained iron oxide nanoparticles had a lint-like form while the synthesized alumina particles were in the form of spheres; hence, the combination of alumina and iron oxide was easy due to their forms. According to Fig. 2a, the average nanoparticle size of the F_10_/γA5 nanocomposite was 76.86 nm with the smallest and largest particles being 40.029, and 112.04 nm, respectively..

Magnetic properties are significantly influenced by particle size, with a notable trend towards the emergence of magnetic behaviors as particle size decreases to the nanometer range. In the context of iron oxides, when the size of magnetic nanoparticles surpasses 50 nm, they are classified as paramagnetic iron oxides. Conversely, particles with sizes smaller than 50 nm fall under the category of ultra-fine superparamagnetic particles. In our investigation, all samples underwent examination using a magnet to facilitate the segregation of nanocomposites from water. VSM results of the nanocomposite powder’s magnetization behavior at room temperature is showcased in Supplementary Fig. S.6. The inset provided a visual representation of the F_10_/γA5 nanocomposite dispersed in water, along with its response to an external magnet. The determined saturation magnetization value for the nanocomposite powder stands at 50.03 emu/g for F_10_/γA5. Due to the significant magnetism exhibited by the material, it can be asserted that the presence of non-magnetic phases in the sample was minimal, if not entirely absent.

### Nitrate removal

#### pH effect

**Figure 3:**
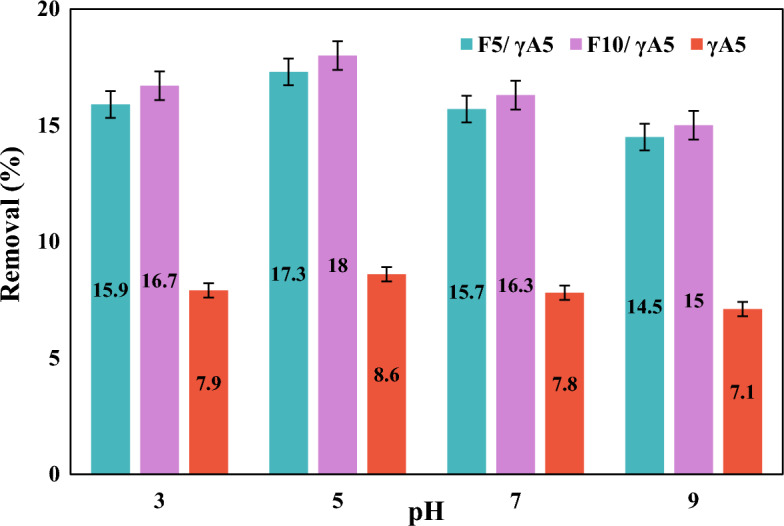
Effect of pH on nitrate removal for 0.1 g of γA5, F_10_/γA5, and F_5_/γA5 at initial nitrate concentration of 10 ppm.

pH is one of the important factors that can play a crucial role in the adsorption process by affecting the structure of pollutants and the charge of the adsorbent’s surface. This study revealed that the pH of the solution had a noticeable effect on the removal percentage. To systematically explore this impact, 0.1 g of each material was introduced into a nitrate solution (10 ppm), and the solution’s pH was adjusted to 3, 5, 7, and 9. As depicted in Fig. 3, the removal of nitrate exhibited higher efficiency at lower pH values, and gradually diminishing as the pH shifted towards alkaline conditions. Specifically, the minimum and maximum nitrate removal values were observed at pH = 9 and pH = 5, respectively. The heightened removal percentage at lower pH values can be rationalized by the simultaneous increase in H^+^ ions and a decrease in OH^−^ ions. This led to a positively charged surface, fostering electrostatic attraction forces between the adsorbent and the negatively charged nitrate^63,64^. Subsequent investigations, based on the obtained results, were conducted at a pH of 5 due to its optimal performance in nitrate removal.

#### Time effect

**Figure 4:**
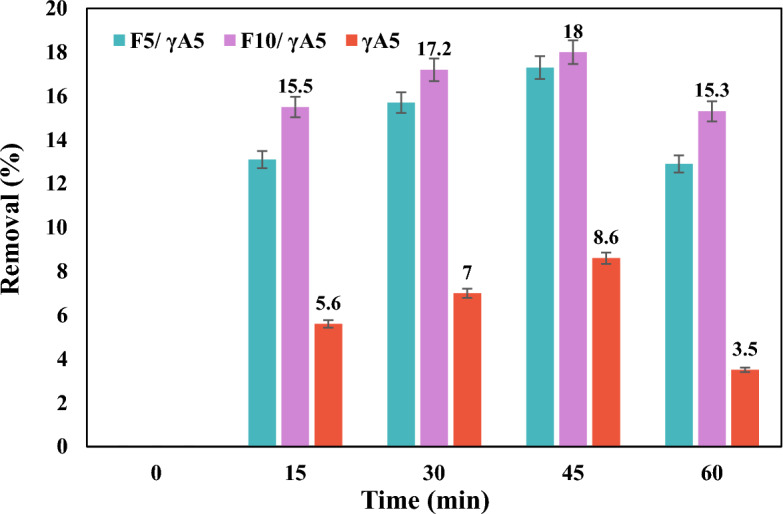
Effect of time on nitrate removal of γA5, F_10_/ γA5, and F_5_/γA5 at initial nitrate concentration of 10 ppm and pH of 5.

In this study, nano γA5 and nanocomposites (F_5_/γA5, F_10_/γA5) were added to a nitrate solution with an initial concentration of 10 ppm and a pH of 5, each at a dosage of 0.1 g per 100 ml. Figure 4 illustrates the impact of contact time on the nitrate removal process by the investigated samples. Notably, the removal percentage for all samples exhibited an initial upward trend within the first hour of the experiment; however, this percentage gradually declined after the 60 min mark. Based on the obtained results, the reaction equilibrium time for γA5, F_5_/γA5, and F_10_/γA5 was determined as 60 min. During the initial phase, active sites on the adsorbent surface were readily available and easily accessible, leading to continuous nitrate adsorption over time. The decrease in nitrate removal percentage following an increase in reaction time can be attributed to several factors inherent in the adsorption process. Initially, active sites on the adsorbent surface are readily available and easily accessible, leading to continuous nitrate adsorption over time. However, as the reaction progresses, several phenomena occur that can lead to a decline in removal efficiency. Firstly, the saturation of active sites plays a significant role. As the reaction proceeds, the active sites on the adsorbent surface become progressively occupied by nitrate ions. Once these sites are saturated, further adsorption becomes limited, resulting in a decrease in removal efficiency. Secondly, mass transfer limitations can impact the process. As the reaction proceeds, a concentration gradient between the solution and the adsorbent surface may develop. This gradient can lead to slower diffusion of nitrate ions from the bulk solution to the active sites on the adsorbent surface, causing a decrease in removal efficiency. Lastly, changes in the adsorbate-adsorbent interaction may occur over time. Factors such as changes in pH, temperature, or the formation of complexes between the adsorbate and the adsorbent material can influence the affinity of nitrate ions for the adsorbent surface, leading to a decrease in removal efficiency. Overall, it was observed that F_10_/γA5 exhibited superior efficiency than other samples.

#### Adsorbent dosage effect

**Figure 5:**
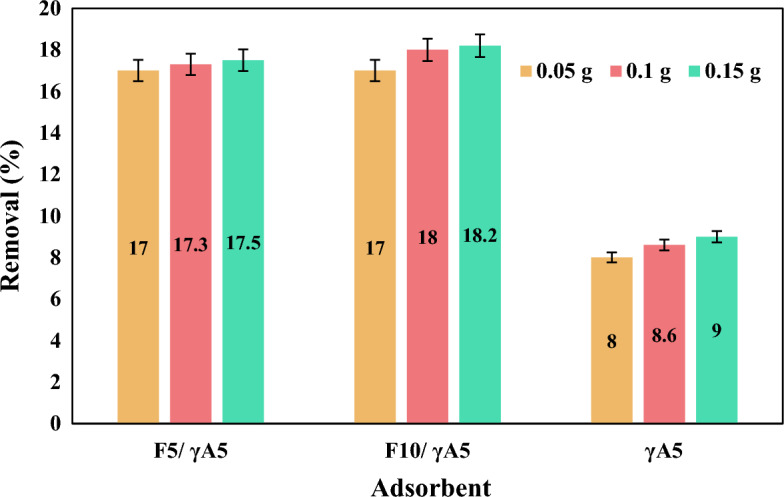
Effect of γA5, F_10_/ γA5, and F_5_/ γA5 dosage on nitrate removal at initial nitrate concentration of 10 ppm and pH of 5.

To assess the impact of adsorbent dosage on nitrate removal (refer to Fig. 5), solutions containing nitrate with an initial concentration of 10 ppm were prepared, and their pH was adjusted to 5. Subsequently, varying amounts of prepared adsorbents, specifically 0.05, 0.1, and 0.15 g in 100 ml, were individually introduced into each solution. Sampling was then conducted at the equilibrium time. The removal percentage exhibited an increasing trend with the augmentation of adsorbent dosage from 0.05 to 0.1 g. However, it was observed that an additional increase in adsorbent dosage to 0.15 g/100 ml did not yield a significant enhancement in efficiency for all samples. Consequently, 0.1 g/100 ml was identified as the optimal dosage for all three samples, striking a balance between effective nitrate removal and resource utilization.

#### Nitrate concentration effect

**Figure 6:**
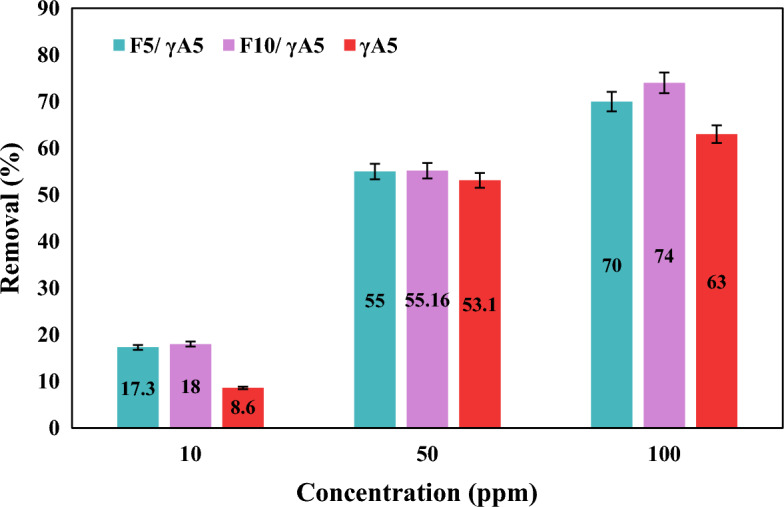
Effect of initial concentration on nitrate removal for 0.1 g of γA5, F_10_/γA5, and F_5_/γA5 at pH of 5.

The influence of nitrate’s initial concentration on the removal percentage was investigated utilizing 0.1 g/100 ml of adsorbents in solutions with initial concentrations of 10, 50, and 100 ppm, all maintained at a pH of 5. Following the attainment of equilibrium conditions, samples were collected and tested for the residual nitrate amount, with the comparative results depicted in Fig. 6. The experimental findings revealed that, at higher nitrate concentrations, the removal percentage for all samples increased. This observed phenomenon can be rationalized by the surplus presence of nitrate ions at higher concentrations, facilitating increased contact between active sites and contaminants. Consequently, the adsorption process became more accessible, leading to enhanced effectiveness in nitrate removal.

To facilitate a comparison between the findings of the current study and those of prior research, a summarized table Table 1 is presented, incorporating comparable results from this study and other relevant studies.

**Table 1:** Comparison of the adsorption capacity and various experimental conditions among multiple adsorbents for nitrate removal.

Material	Nitrate Removal (%)	Conditions	References
F_10_/γA5	74	Initial concentration = 100 ppm, pH = 5, contact time = 45 min	This study
γalumina	$\sim$ 68	Initial concentration = 20 ppm, pH = 6.68, contact time = 30 min	^47^
Zn/Al chloride layered double hydroxide	85	Initial concentration = 10 ppm, pH = 6, contact time = 40 min	^65^
Fe_3_O_4_/MB	79	Initial concentration = 30 ppm, pH = 7.5, contact time = 90 min	^63^
Fe-Zeolite chitosan beads	89	Initial concentration = 50 ppm, pH = 5, contact time = 60 min	^58^
FeMgMn-LDH,	$\sim$ 61	Initial concentration = 20 ppm, pH = 7, contact time = 4 h	^66^
Alumina magnetic hybrid	$\sim$ 50	Initial concentration = 100 ppm, pH = 6, contact time = 80 min	^67^
Al_2_O_3_/ZrO_2_/Fe_3_O_4_	50	Initial concentration = 15 ppm, pH = 3, contact time = 24 h	^68^

#### Kinetic study

Enhancing our comprehension of adsorption processes, specifically pore-diffusion and surface-adsorption, can be facilitated through the application of kinetic models. These models not only provide insights into the adsorption mechanisms but also offer valuable information on the controlling factors, such as mass transfer, diffusion control, and chemical reaction. The kinetics of adsorption play a pivotal role in determining optimal operating conditions for full-scale batch processes, and they provide crucial information for modeling and designing the overall process. In this study, we employed pseudo-first-order, pseudo-second-order, and intraparticle diffusion kinetic models, as described by Eqs. (4–6), respectively.

4 $${{\text{q}}}_{{\text{t}}}={{\text{q}}}_{{\text{e}}}(1-{{\text{e}}}^{{{\text{k}}}_{1}{\text{t}}})$$

5 $$\frac{{\text{t}}}{ {{\text{q}}}_{{\text{t}}}}=\frac{1}{{{\text{k}}}_{2}{{\text{q}}}_{{\text{e}}}^{2}}+\frac{{\text{t}}}{{{\text{q}}}_{{\text{e}}}}$$

6 $${{\text{q}}}_{{\text{t}}}={{\text{k}}}_{3}{{\text{t}}}^{0.5}+{\text{C}}$$

Adsorption capacity at equilibrium and the amount of adsorbed nitrate at the contact time of t (min) showed by *q*_*e*_ (mg/g) and *q*_*t*_ (mg/g). Constant rates of pseudo-first-order, pseudo-second-order, and Weber and Morris (intraparticle diffusion) models are attributed to *k*_1_ (L/min), *k*_2_ (g/mg min), and *k*_3_ (g/mg min^0.5^). C (mg/g) represents the thickness and boundary layer and *R*^2^ stands for the regression factor^69,70^. All the derived parameters are tabulated in Table 2. It is noteworthy that all tested materials exhibited a good fit with both the Pseudo-second-order and Intra-particle diffusion kinetic models. The adherence to the Pseudo-second-order model suggested that chemisorption acts as the rate-limiting step in the adsorption process. This implied that the rate of adsorption was primarily dependent on the adsorption capacity rather than the concentration of nitrate. The Intra-particle diffusion model predicated on the assumption that the slowest step in the adsorption process was associated with the nitrate (adsorbate) in a liquid film surrounding the adsorbent. The well-established compatibility of the materials with this model indicated the likelihood of intra-particle diffusion playing a significant role in the adsorption mechanism. This underscored the potential involvement of intra-particle diffusion in governing the overall adsorption process, as supported by the model’s favorable alignment with the experimental data^71^.

**Table 2:** Obtained kinetic parameters for γA5, F_5_/ γA5, and F_10_/γA5.

Kinetic model	Parameters	γA5	F_5_/ γA5	F_10_/γA5
Pseudo-first-order	k_1_ (L/min)	0.0928	0.0944	0.1024
	q_e_ (mg/g)	65.3658	67.0005	77.4785
	R^2^	0.8912	0.8732	0.8875
Pseudo-second-order	k_2_ (g/mg min)	0.0397	0.0625	0.1197
	q_e_ (mg/g)	63.6943	70.9220	74.6269
	R^2^	0.9517	0.9849	0.9863
Intra-particle diffusion	k_3_ (g/mg min^0.5^)	3.6616	6.0204	7.6928
	C (mg/g)	10.582	29.954	49.873
	R^2^	0.9871	0.9963	0.9837

## Rationale and involved mechanisims

Current methods for removing nitrate from drinking water include diverse techniques like ion exchange resin, biological denitrification, chemical denitrification, electrodialysis, reverse osmosis, and catalytic denitrification. However, each of these approaches comes with its own set of limitations. The regeneration process for ion-exchange resin complicates and increases the cost of the procedure. Biological denitrification introduces concerns about organic contamination and excessive chlorine usage. The use of chemicals in chemical denitrification raises safety issues, leads to side reactions, and adds to the overall expenses, making it economically impractical. Electrodialysis is not a straightforward and cost-effective method either. Despite being energy-intensive and having low throughput, reverse osmosis is not a suitable option. Finally, catalytic denitrification becomes economically unfeasible due to the involved pre- and post-treatment steps, along with the additional cost of chemicals^72^. Among the various applications of nanotechnology in the field of environmental science, the remediation of polluted groundwater using iron oxide nanoparticles has garnered considerable attention due to their exceptional adsorptive capabilities towards important water contaminants^73^.

The interaction between aluminum oxide (Al_2_O_3_) and magnetite (Fe_3_O_4_) primarily involves surface interactions, as these compounds do not typically engage in direct chemical bonding. Surface forces such as Van der Waals forces, electrostatic attractions or repulsions, and the presence of specific chemical functionalities play a role in their interaction. Van der Waals forces, encompassing dipole–dipole interactions and London dispersion forces, may contribute to attractions between the surfaces due to slight charge imbalances. Additionally, electrostatic forces arising from the differing charges on the surfaces could lead to attractive interactions. The presence of surface functional groups, such as hydroxyl groups on Al_2_O_3_ and metal–oxygen groups on magnetite, may facilitate interactions through mechanisms like hydrogen bonding or coordination bonds. Adsorption phenomena, where molecules from the surrounding environment adhere to the surfaces, could further contribute to the adherence between Al_2_O_3_ and magnetite^74^.

The presence of metal ions in adsorbents often contributes to the electrostatic attraction. This is because metal oxides typically form hydrated metal oxides (M–OH) in aqueous media, and under strongly acidic conditions, protonated metal oxides generate a positively charged adsorbent surface, enabling them to interact with nitrate through electrostatic attraction. The adsorption of nitrate ions onto certain adsorbents containing surface functional groups, such as hydroxyl, amine, and carboxyl groups, may involve electrostatic attraction, and these adsorbents become protonated under strongly acidic conditions^19^.

Therefore, the adsorption of nitrate by synthetic inorganic nanocomposites is considered a noteworthy approach due to its proven effectiveness, efficiency, cost-effectiveness, and simplicity, as well as its ability to overcome the issues encountered with other conventional water purification methods. Magnetic separation technology plays a significant role in the development of magnetite-based nanocomposites, offering great potential for treating large volumes of wastewater and simplifying the separation process by using an external magnet. To enhance the adsorption performance of Fe_3_O_4_ for ions, other elements such as Mn, Al, Ce, and Zr oxides can be incorporated. Thus, various composite adsorbents incorporating magnetite have been synthesized, as the addition of Fe_3_O_4_ facilitates easy separation of the adsorbent from the sample solution using a magnet, and also allows for modifications of functional groups to target specific contaminants^68^. The interaction between nitrate and metal oxide can be described as a two-step ligand exchange reaction involving metal ions (Eqs. 7 and 8). Al^3+^ in the composites can be four, five and six corrdinated. The corrdination number of Al_2_O_3_ is normally four, that three of them can be placed on a plane. In the Eqs. 7 and 8, two lines attached to M relate the cation’s substituents. Also, the synthesized composites have OH groups. So, the nitrate can be adsorbed by ion exchange or through hydrogen bonding with hydroxyl groups as illustrated in Eq. 9 and 10. The pH influence was studied for removing nitrate. So, in the low pH like 3, the OH groups on the composite could be protonated. This event made the electrostatic attraction between adsorbent and nitrate dscribed by Eq. 11^2,68^. The whole mechanism and binding are briefly described as follows:

7 $$\left. {} \right\rangle MOH + H^{ + } \rightleftharpoons MOH_{2}^{ + }$$

8 $$\left. {} \right\rangle MOH_{2}^{ + } + NO_{3}^{ - } \rightleftharpoons \left. {} \right\rangle MOH_{2} - NO_{3} \left( {or MNO_{3} + H_{2} O} \right)$$

9 $$M-OH+ {NO}_{3}^{-}\to M-N{O}_{3}^{-}+ {OH}^{-}$$

10 $$M-OH+{NO}_{3}^{-}\to M-O{H}_{\cdot } \dots {NO}_{3}^{-}$$

11 $$M-{OH}_{2}^{+}+ {NO}_{3}^{-}\to {M-OH}_{2\cdot }^{+}\dots N{O}_{3}^{-} {\left(M:Al,Fe\right)}^{68}$$

## Conclusion and future considerations

In this study, γ-alumina nanoparticles were successfully synthesized using five distinct methods (γA1-5), with the γA2 and γA5 samples demonstrating exceptional alignment with the standard γ-alumina sample. Furthermore, nanocomposites, denoted as F_n_/γA5, were developed by incorporating Fe_3_O_4_ nanoparticles with varying weight percentages (denoted as n). The confirmation of successful synthesis was obtained through comprehensive analyses employing XRD, FT-IR, FESEM, EDX, XRD, and VSM techniques.

Crucial factors influencing the adsorption performance of the prepared nanocomposites were systematically investigated. The optimal adsorption conditions were identified by examining the effects of pH, contact time, adsorbent dosage, and initial nitrate concentration. Among the nanocomposites, F_10_/γA5 demonstrated superior adsorption capacity. The conducted experiments for γA5 and F_5_/γA5, F_10_/γA5 nanocomposites showcased the optimum pH of 5 and contact time of 45 min for all samples. Increasing of adsorbent dosage from 0.05 to 0.1 g, resulted in a rise in nitrate removal percentage for γA5 at 100 ppm, progressing from 8 to 8.6%. Similarly, for F_5_/γA5 and F_10_/γA5, the percentage increased from 17 to 17.3 and 17 to 18, respectively. Further increase of adsorbents to 0.15 g resulted in negligible increase of 0.2%; hence, 0.1 g was chosen as the optimum dosage. The influence of nitrate’s initial concentration on the removal percentage was investigated with initial concentrations of 10, 50, and 100 ppm. γA5 and nanocomposites F_5_/γA5, F_10_/γA5 had nitrate removal efficiency of 17.3, 55, and 70% at 10 ppm, 18, 55.16, and 74% at 50 ppm, and 8.6, 53.1, and 63%, respectively. It is noteworthy that all tested materials exhibited a good fit with both the Pseudo-second-order and Intra-particle diffusion kinetic models.

In conclusion, the synthesized γ-alumina nanoparticles and magnetic nanocomposites exhibited promising characteristics for efficient pollutant removal from water sources, thereby showcasing their potential in addressing environmental challenges. The comprehensive understanding of their synthesis, characterization, and adsorption performance contributes valuable insights to the field and underscores their applicability in water treatment processes.

Regarding the future outlook, the surface adsorption method for nitrate removal in large-scale aquifers can be considered, especially for treating industrial wastewater and rural groundwater. This is because the filtration process is challenging and costly. However, the surface adsorption method on alumina and magnetite is suitable since it is less expensive and provides multiple operational cycles for us by applying a magnetic field. Moreover, recycleability and reusability of the materials should be assessed.

### Supplementary Information

Supplementary Information.

## Data Availability

All data generated or analysed during this study are included in this published article and its supplementary information files.
