# Supplementary material for: Nitrate removal study of synthesized nano γ-alumina and magnetite-alumina nanocomposite adsorbents prepared by various methods and precursors
Source: Sci Rep. 2024 Apr 1;14:7673. doi: 10.1038/s41598-024-58459-z (PMC10984990; doi:10.1038/s41598-024-58459-z)
Supplement: Supplementary file 1 — Supplementary Information. [file 41598_2024_58459_MOESM1_ESM.docx]

## Materials and instruments

Nepheline Syenite (>90%), and Lime (>90%) were obtained from *Azarshahr*, located in *East Azerbaijan*, *Iran*. Aluminum hydroxide (Al(OH)_3_) was provided from *Jajarm*, located in *North Khorasan*, *Iran*. Polyethylene glycol (C_2n_H_4n+2_O_n+1_), Sodium hydroxide (NaOH), Nitric acid (HNO_3_), Iron (II) chloride tetrahydrate (FeCl_2_ .4H_2_O), Iron (III) chloride hexahydrate (FeCl_3_.6H_2_O), and Potassium nitrate (KNO_3_) were purchased from *Merck*. Hydrochloric Acid (HCl), Ethanol (C_2_H_6_O) (98%), and Ammonia (NH_3_, 25%) were acquired from *Royalex*, *JATA co.*, and *Fakhre Razi*, sequentially. All of the synthesized materials were scrutinized using Field emission scanning electron microscopy (FESEM; TESCAN Mira III), Fourier transform infrared spectroscopy (FTIR; Brucker Model Tensor27), X-ray diffraction analysis (XRD; D500 Siemens), and BET (Nanosoord). XRD of the materials was recorded within the range of 2θ= 4°-70° with Cu Kα2 (54.1 Å) radiation, which were meticulously scrutinized using X’Pert HighScore Plus software. The concentration of Nitrate was investigated using UV-visible absorption spectra (UV-Vis, SPECORD 40). Detecting the magnetic properties of the nanocomposite powder at room temperature involved the use of a Model 7400 VSM, with a maximum applied field reaching 20,000 G (2 T).


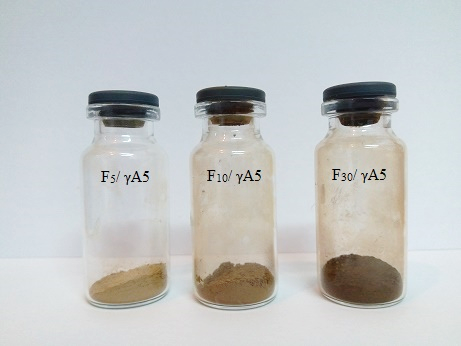


F_5_/γA5

F_10_/γA5

F_30_/γA5

Fig.S.1. Magnetite/γ-Alumina nanocomposites.

Fig.S.2. Nitrate standard curve.


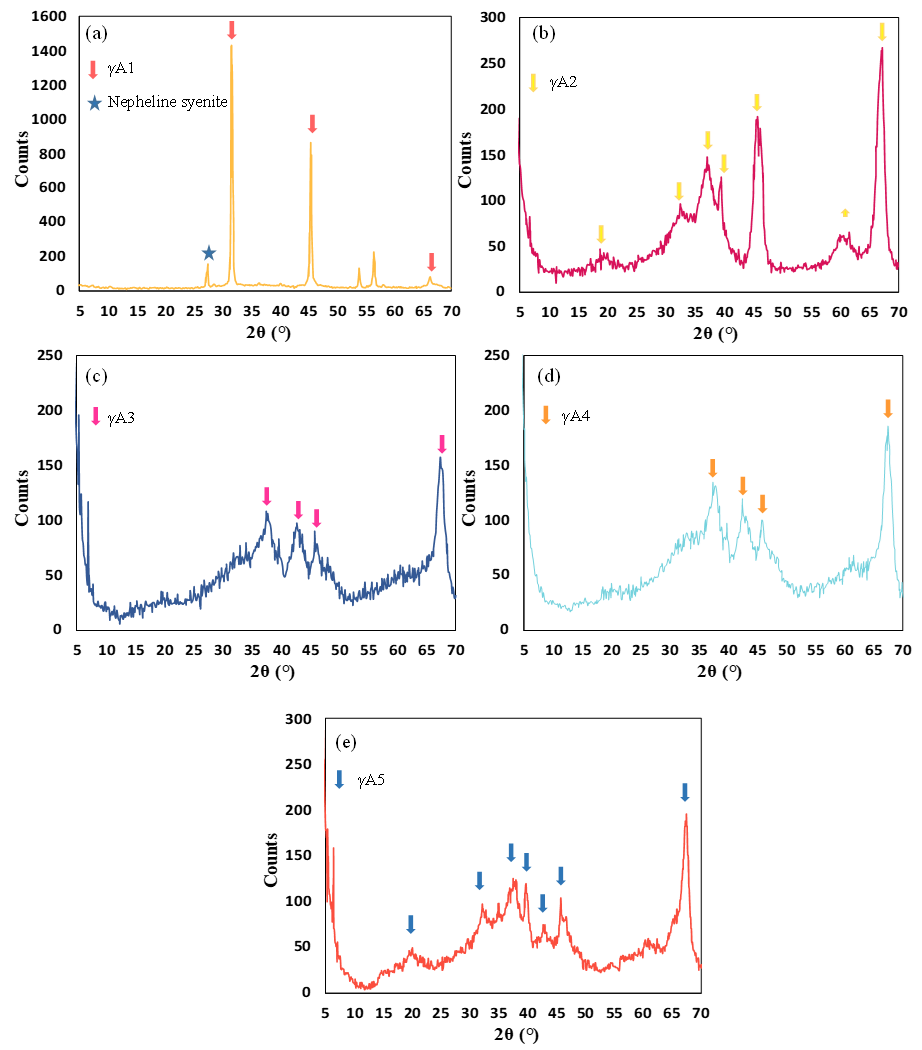


Fig.S.3. XRD pattern of (a) γA1, (b) γA2, (c) γA3, (d) γA4, and (e) γA5.


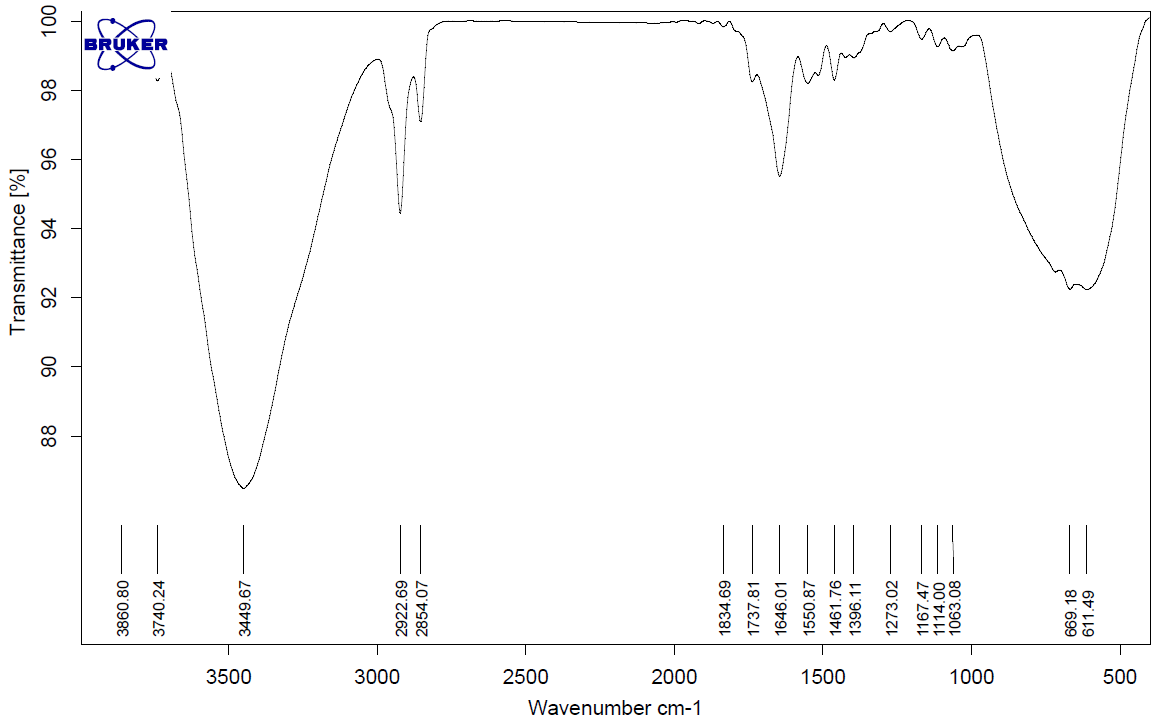


Alumina formation

Al-O-Al

O-H

O-H


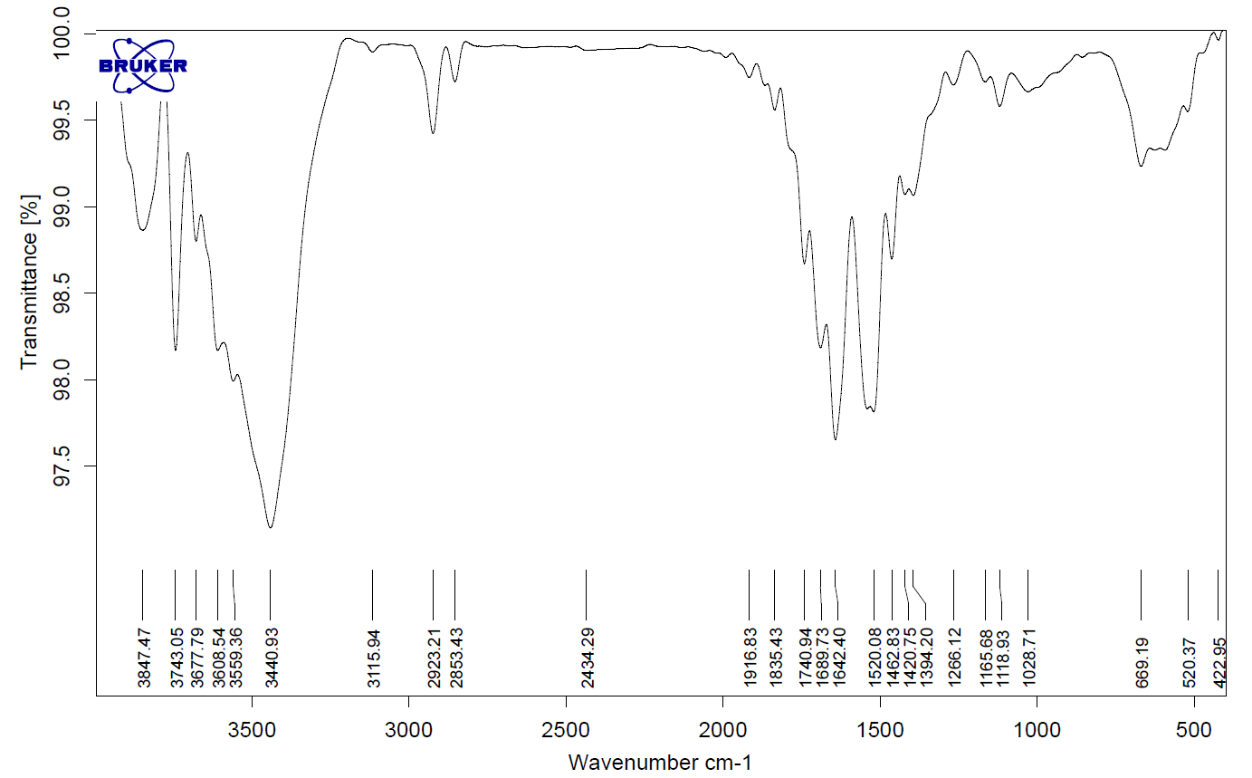


Al-O-Al

Alumina formation

O-H

O-H

(a)

(b)


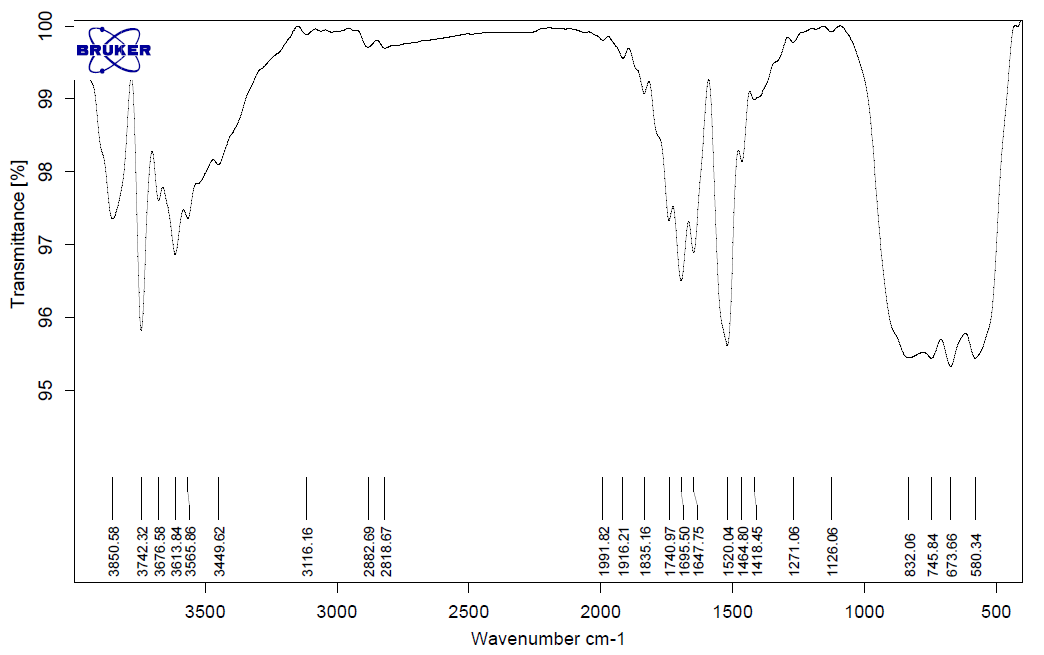


Al-O-Al

Alumina formation

O-H

O-H


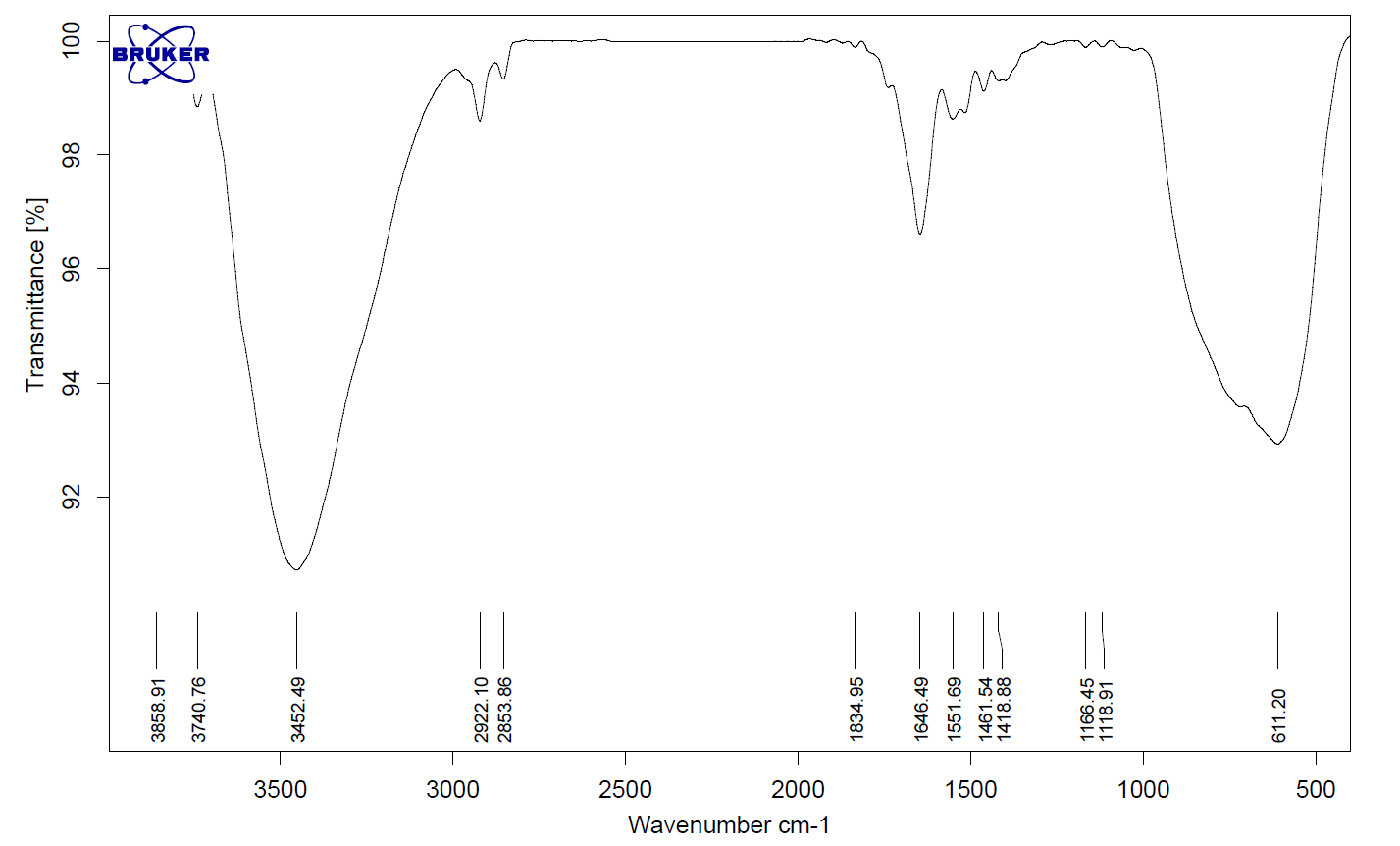


O-H

O-H

Al-O-Al

Alumina formation

(c)

(d)

Fig.S.4. FT-IR spectra of (a) γA2, (b) γA3, (c) γA4, and (d) γA5.


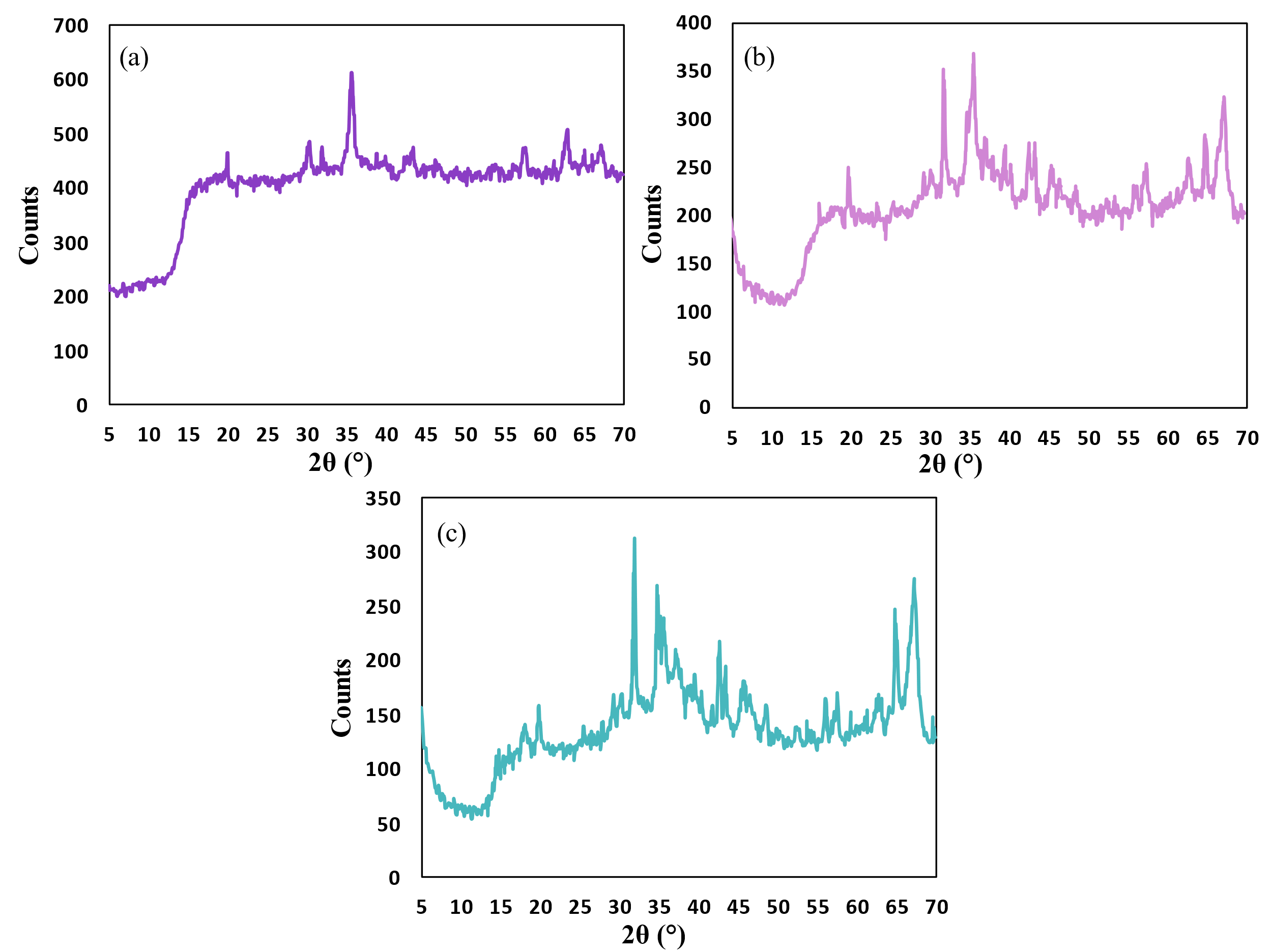


Fig.S.5. XRD pattern of (a)F_30_/ γA5, (b) F_10_/ γA5, and (c) F_5_/ γA5.


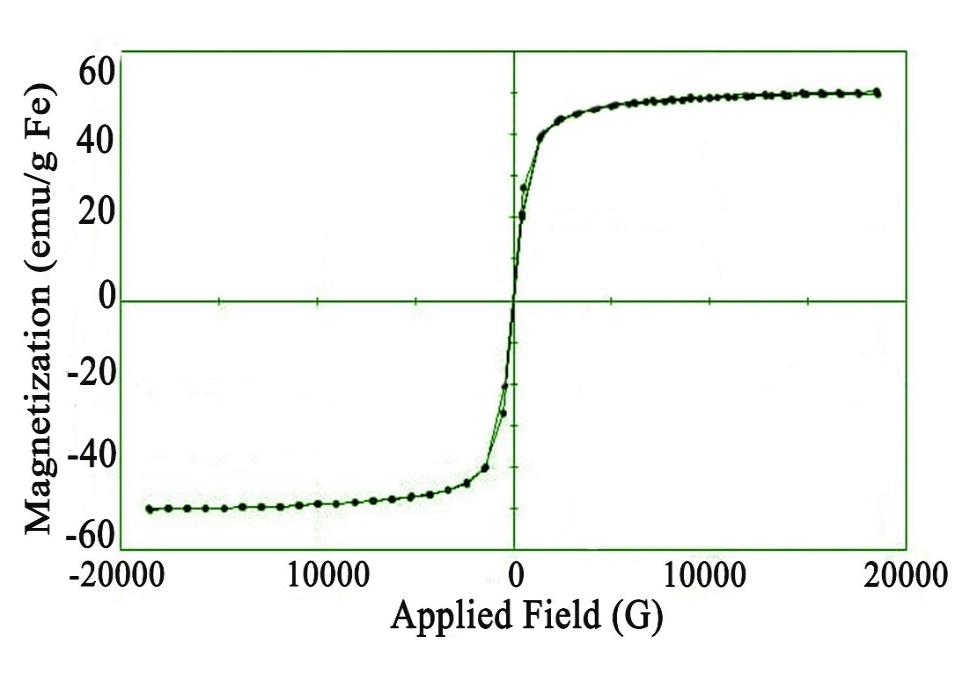


Fig. S.6. VSM graph for F_10_/ γA5.

Table S.1. characteristic of standard Alumina.

| **Name and formula**  Reference code: 01-075-0921  ICSD name: Aluminum Oxide  Empirical formula: Al_2.666_O_3.999_  Chemical formula: (Al_2_O_3_)_1.333_  **Crystallographic parameters**  Crystal system: Cubic  Space group: Fm-3m  Space group number: 225  a: 3/9500  b: 3/9500  c: 3/9500  Alpha (°): 90/0000  Beta (°): 90/0000  Gamma (°): 90/0000  Calculated density (g/cm^3): 3/66  Volume of cell (10^6 pm^3): 61/63  Z: 1/00  RIR: 1/09  **Subfiles and Quality**  Subfiles: Inorganic  Alloy, metal or intermetalic  Corrosion  Modelled additional pattern  Quality: Calculated (C)  **Comments**  ICSD collection code: 030267  Test from ICSD: No R value given.  At least one TF missing.  **References**  Primary reference: *Calculated from ICSD using POWD-12++*, (1997)  Structure: Verwey, E.J.W., *Z. Kristallogr., Kristallgeom., Kristallphys., Kristallchem.*, **91**, 317, 1935  **Peak list**  No. h k l d [A] 2Theta[deg] I [%]  1 1 1 1 2.28053 39.482 4.9  2 2 0 0 1.97500 45.912 100  3 2 2 0 1.39654 66.951 94.2  4 3 1 1 1.19097 80.599 0.1  5 2 2 2 1.14027 84.992 9.4 |
| --- |
| **Name and formula**  Reference code: 00-001-1303  PDF index name: Aluminum Oxide  Empirical formula: Al2O3  Chemical formula: Al2O3  **Crystallographic parameters**  Crystal system: Cubic  a: 3.4100  b: 3.4100  c: 3.4100  Alpha (°): 90.0000  Beta (°): 90.0000  Gamma (°): 90.0000  Measured density (g/cm^3): 3.47  Volume of cell (10^6 pm^3): 39.65  RIR: -  **Status, subfiles and quality**  Status: Marked as deleted by ICDD  Subfiles: Inorganic  Alloy, metal or intermetallic  Quality: Doubtful (O)  **Comments**  Deleted by: Deleted by 10-425 (JVS August 27, 1959).  Color: Colorless  General comments: Low temperature.  Aluminum Research Lab.  Transforms to hexagonal form at 750-1000 C.  Optical data: B=1.696  **References**  Primary reference: Aluminum Co. of America, New Kensington, PA, USA., *Private Communication*  Optical data: *Data on Chem. for Cer. Use, Natl. Res. Council Bull. 107*  **Peak list**  No. h k l d [A] 2Theta[deg] I [%]  1 1 1 0 2.39000 37.604 19.0  2 1 1 1 1.98000 45.790 72.0  3 2 1 0 1.52000 60.899 6.0  4 2 1 1 1.40000 66.763 100.0  5 3 0 0 1.14000 85.017 13.0  6 2 2 2 0.99000 102.170 6.0  7 3 3 0 0.81000 143.974 6.0 |
| **Name and formula**  Reference code: 00-004-0880  Common name: alumina  PDF index name: Aluminum Oxide  Empirical formula: Al2O3  Chemical formula: Al2O3  **Crystallographic parameters**  Crystal system: Cubic  Space group: P  a: 7/9500  b: 7/9500  c: 7/9500  Alpha (°): 90/0000  Beta (°): 90/0000  Gamma (°): 90/0000  Volume of cell (10^6 pm^3): 502/46  Z: 10/00  RIR: -  **Subfiles and Quality**  Subfiles: Inorganic  Alloy, metal or intermetalic  Cement and Hydration Product  Corrosion  Forensic  Superconducting Material  Quality: Blank (B)  **Comments**  General comments: d and I value revised by Stumpf in 1960, using corundum as standard.  All reflections are diffuse.  Sample preparation: α alumina trihydrate heated 1 hour at 800 C in moving dry air.  **References**  Primary reference: Stumpf et al., *Ind. Eng. Chem*, **42**, 1398, 1950  **Peak list**  No. h k l d [A] 2Theta[deg] I [%]  1 3 1 1 2.40000 37.442 40.0  2 2 2 2 2.27000 39.673 20.0  3 3 2 1 2.11000 42.824 30.0  4 4 0 0 1.98000 45.790 20.0  5 5 1 1 1.53000 60.459 10.0  6 4 4 1 1.39000 67.703 100.0 |

Table S.2. XRD characteristics of γA1, γA2, γA3, γA4, and γA5.

| Material | 2θ | FWHM(2θ) | I |
| --- | --- | --- | --- |
| γA1 | 7.31  27.42  28.61  31.75  36.39  40.19  45.51  53.92  56.51  58.13  66.28 | 0.40  0.16  0.40  0.18  0.20  0.80  0.20  0.23  0.26  0.26  0.40 | 14  112  15  1199  12  16  740  95  166  15  46 |
| γA2 | 6.74  32.62  39.41  43.61  45.69  46.24  66.91 | 1.60  1.33  0.53  0.60  0.53  1.87  1.63 | 23  10  40  12  46  26  124 |
| γA3 | 5.23  5.62  6.84  67.55 | 0.05  0.05  0.06  1.30 | 53  19  46  16 |
| γA4 | 22.94  42.41  45.81  67.30 | 1.33  1.87  0.53  1.30 | 13  10  13  23 |
| γA5 | 5.36  6.43  32.12  39.60  45.63  67.19 | 0.05  0.20  0.80  0.66  0.40  0.81 | 19  59  15  16  24  61 |

Table S.3. XRD characteristics of γA5 and nanocomposites.

| Material | Alumina d values (nm) | Iron d values (nm) | 2θ | FWHM(2θ) | D (nm) |
| --- | --- | --- | --- | --- | --- |
| γA5 | d_1_= 2.78  d_2_= 2.27  d_3_= 1.98  d_4_= 1.39 | - |  |  |  |
| F_5_/ γA5 | d_1_= 2.80  d_2_= 2.27  d_3_= 2.08  d_4_= 1.39 | d_1_= 4.46  d_2_= 2.96  d_3_= 2.52  d_4_=1.64  d_5_=1.60  d_6_=1.43 | γA5= 67.20 | 0.65 | 14.56 |
|  |  |  | Fe_3_O_4_= 31.92 | 0.20 | 40.90 |
| F_10_/ γA5 | d_1_= 2.80  d_2_= 2.27  d_3_= 2.08  d_4_=1.39 | d_1_= 4.46  d_2_= -  d_3_= 2.52  d_4_= 1.63  d_5_= 1.60  d_6_= 1.43 | γA5= 67.20  Fe_3_O_4_= 31.89 | 0.48 | 19.71  35.56 |
|  |  |  |  | 0.23 |  |
| F_30_/ γA5 | d_1_= 2.95  d_2_= -  d_3_= -  d_4_=1.47 | d_1_= 4.45  d_2_= 2.95  d_3_= 2.51  d_4_= -  d_5_= -  d_6_= - | γA5= 62.84 | 0.48 | 19.24 |
|  |  |  | Fe_3_O_4_= 35.64 | 0.46 | 17.97 |
